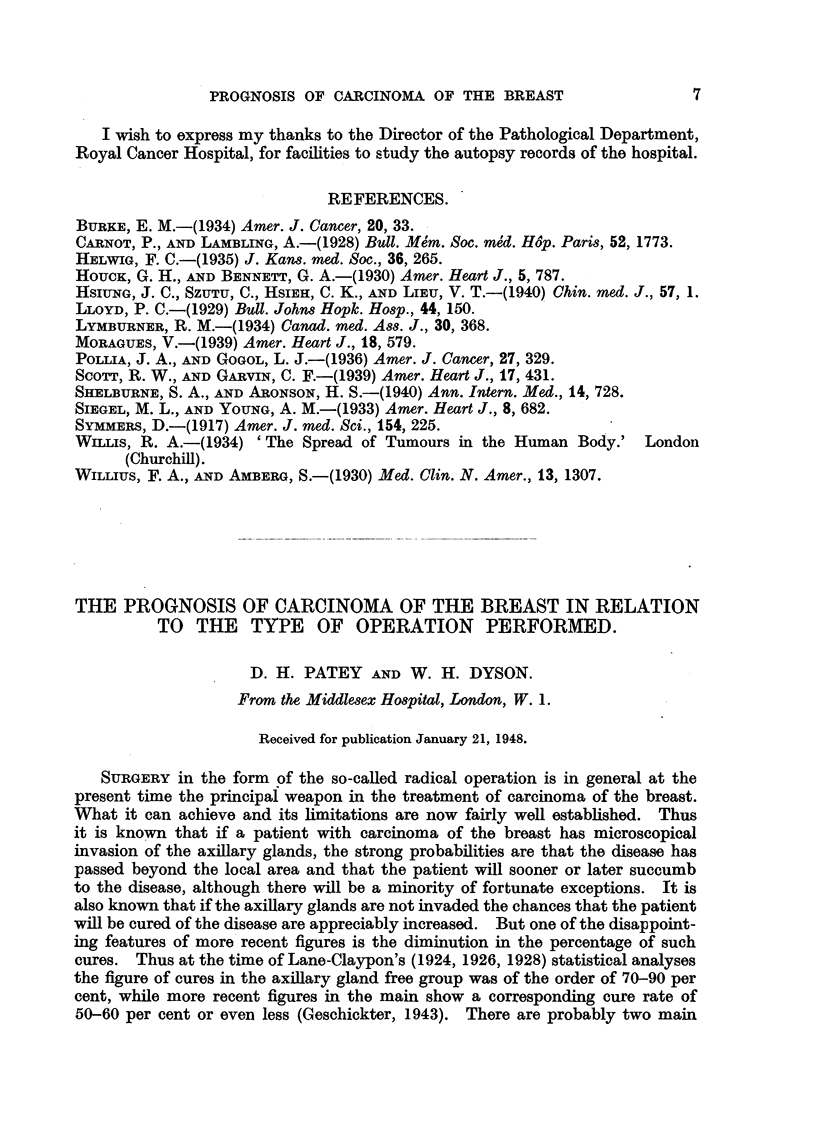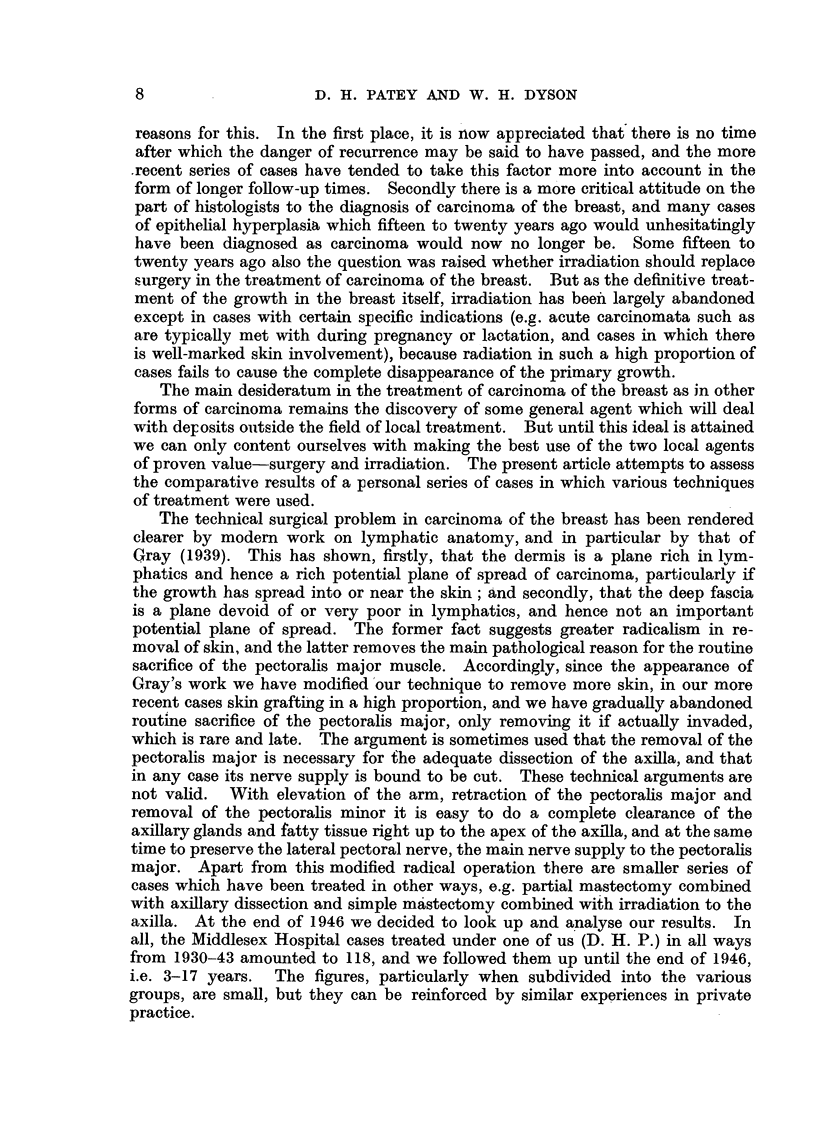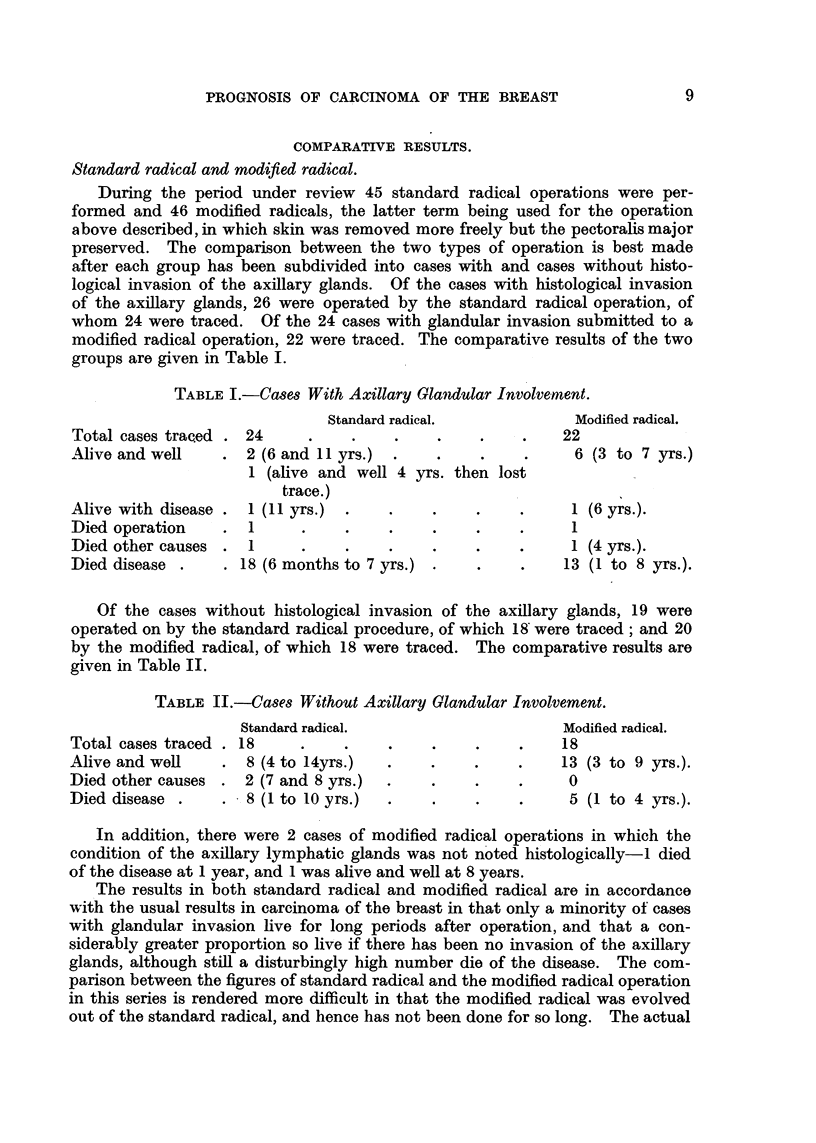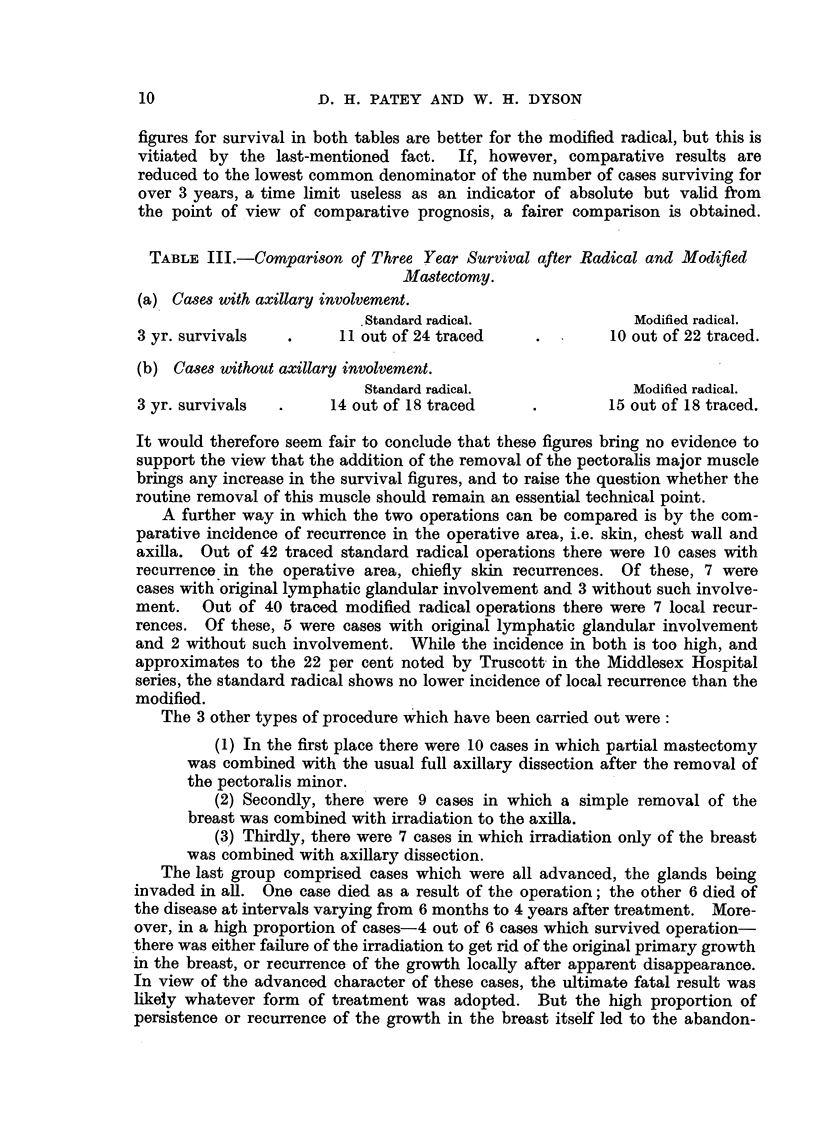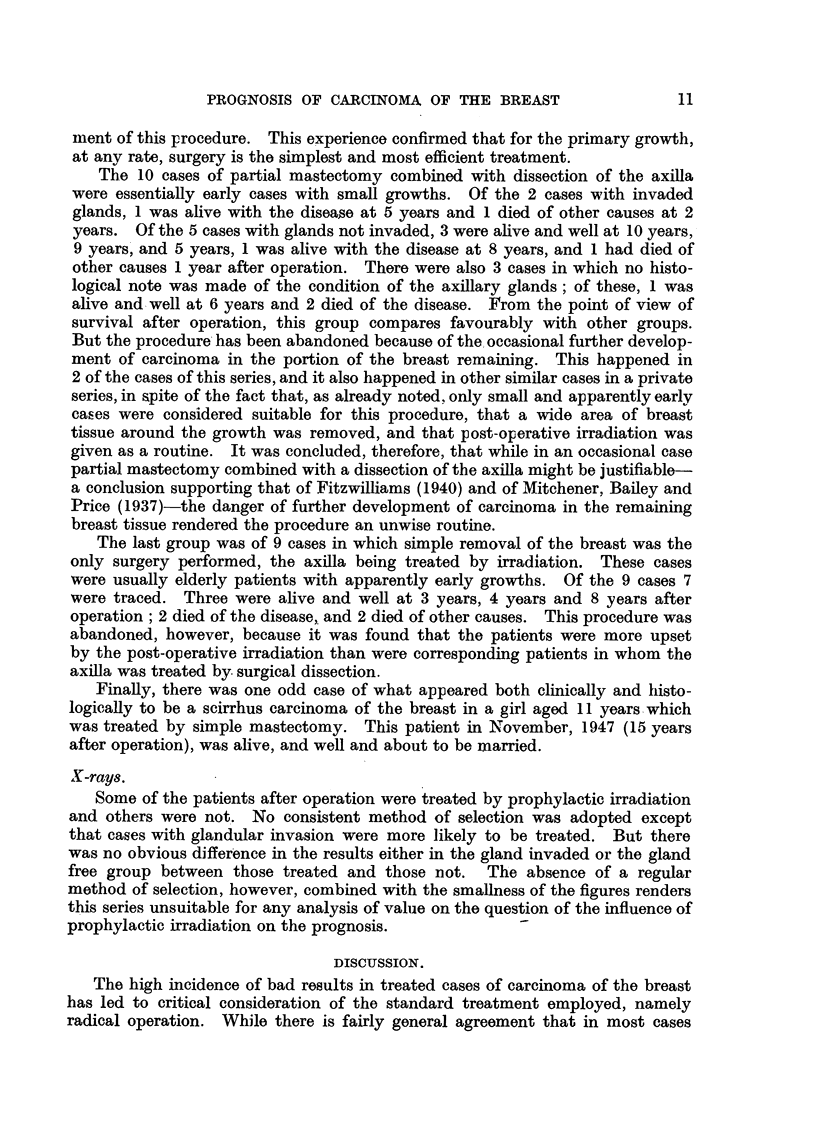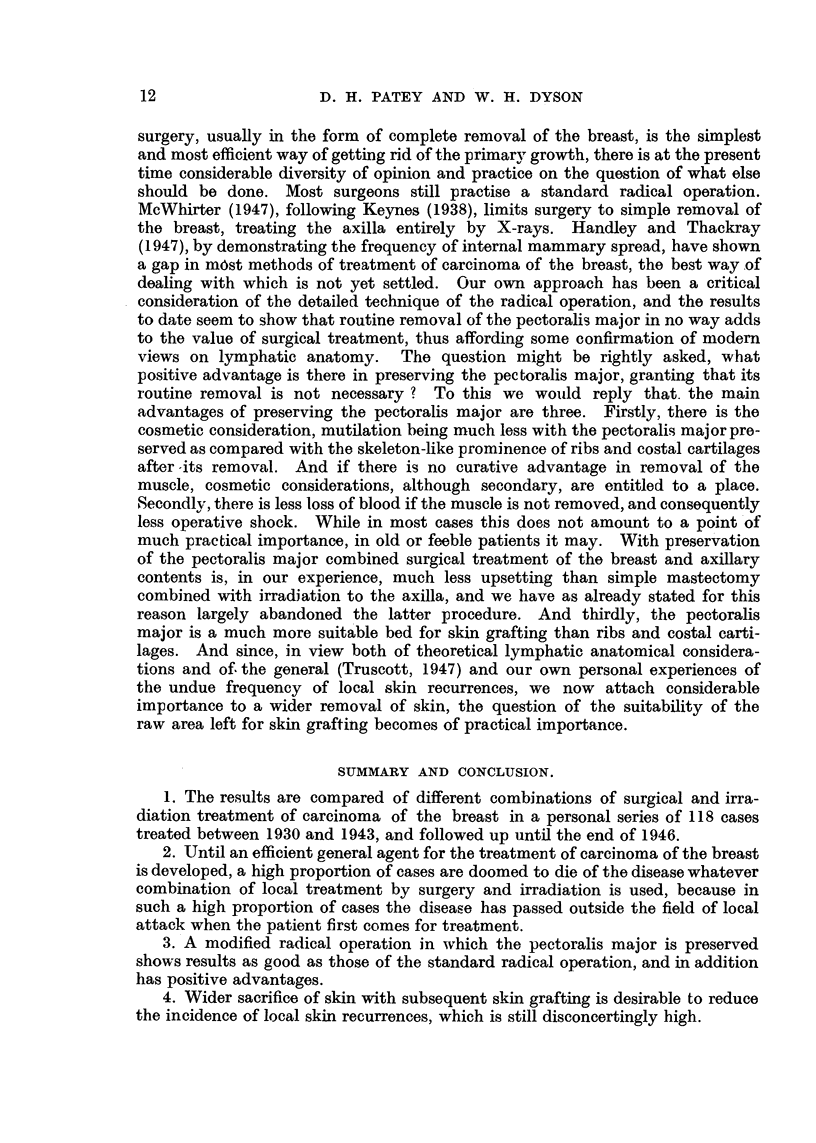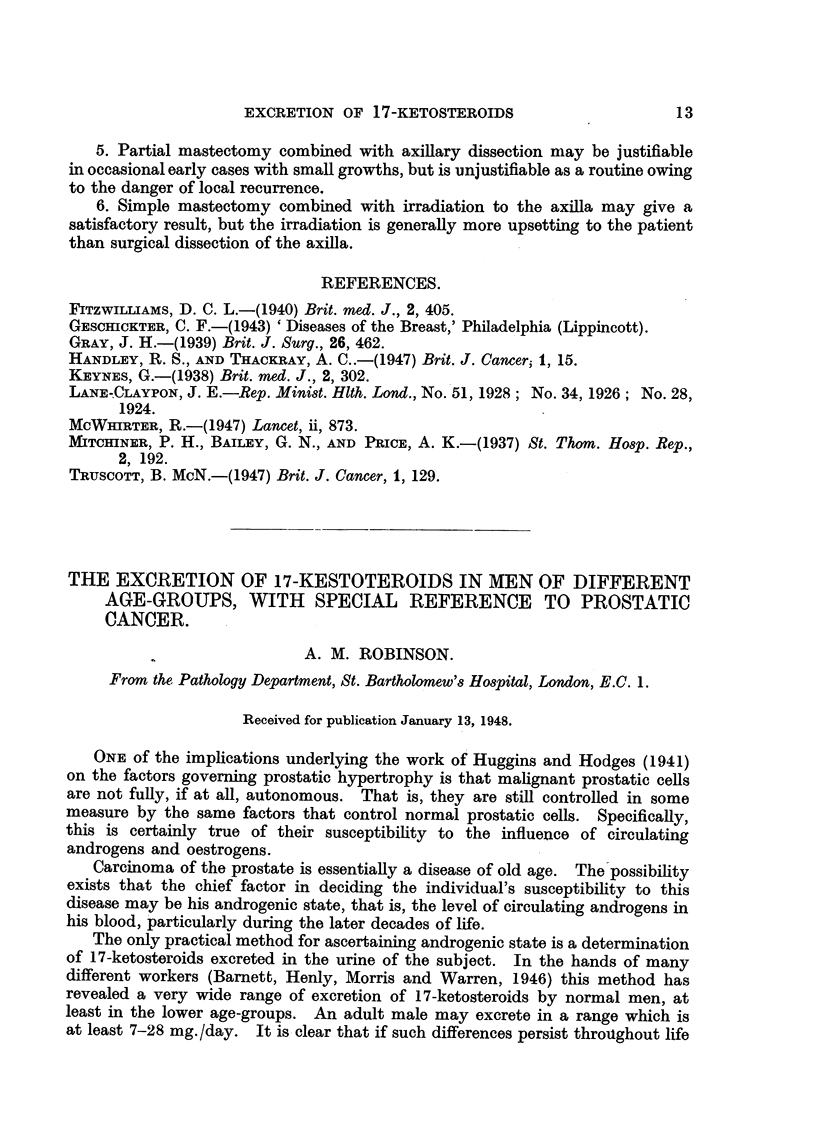# The Prognosis of Carcinoma of the Breast in Relation to the Type of Operation Performed

**DOI:** 10.1038/bjc.1948.2

**Published:** 1948-03

**Authors:** D. H. Patey, W. H. Dyson


### REFERENCES.

### THE PROGNOSIS OF CARCINOMA OF THE BREAST IN RELATION TO THE TYPE OF OPERATION PERFORMED.

D. H. PATEY AND W. H. DYSON.

*From the Middlesex Hospital, London, W. 1.*

Received for publication January 21, 1948.

SURGERY in the form of the so-called radical operation is in general at the present time the principal weapon in the treatment of carcinoma of the breast. What it can achieve and its limitations are now fairly well established. Thus it is known that if a patient with carcinoma of the breast has microscopical invasion of the axillary glands, the strong probabilities are that the disease has passed beyond the local area and that the patient will sooner or later succumb to the disease, although there will be a minority of fortunate exceptions. It is also known that if the axillary glands are not invaded the chances that the patient will be cured of the disease are appreciably increased. But one of the disappointing features of more recent figures is the diminution in the percentage of such cures. Thus at the time of Lane-Claypon's (1924, 1926, 1928) statistical analyses the figure of cures in the axillary gland free group was of the order of 70–90 per cent, while more recent figures in the main show a corresponding cure rate of 50–60 per cent or even less (Geschickter, 1943). There are probably two main reasons for this. In the first place, it is now appreciated that there is no time after which the danger of recurrence may be said to have passed, and the more recent series of cases have tended to take this factor more into account in the form of longer follow-up times. Secondly there is a more critical attitude on the part of histologists to the diagnosis of carcinoma of the breast, and many cases of epithelial hyperplasia which fifteen to twenty years ago would unhesitatingly have been diagnosed as carcinoma would now no longer be. Some fifteen to twenty years ago also the question was raised whether irradiation should replace surgery in the treatment of carcinoma of the breast. But as the definitive treatment of the growth in the breast itself, irradiation has been largely abandoned except in cases with certain specific indications (e.g. acute carcinomata such as are typically met with during pregnancy or lactation, and cases in which there is well-marked skin involvement), because radiation in such a high proportion of cases fails to cause the complete disappearance of the primary growth.

The main desideratum in the treatment of carcinoma of the breast as in other forms of carcinoma remains the discovery of some general agent which will deal with deposits outside the field of local treatment. But until this ideal is attained we can only content ourselves with making the best use of the two local agents of proven value—surgery and irradiation. The present article attempts to assess the comparative results of a personal series of cases in which various techniques of treatment were used.

The technical surgical problem in carcinoma of the breast has been rendered clearer by modern work on lymphatic anatomy, and in particular by that of Gray (1939). This has shown, firstly, that the dermis is a plane rich in lymphatics and hence a rich potential plane of spread of carcinoma, particularly if the growth has spread into or near the skin ; and secondly, that the deep fascia is a plane devoid of or very poor in lymphatics, and hence not an important potential plane of spread. The former fact suggests greater radicalism in removal of skin, and the latter removes the main pathological reason for the routine sacrifice of the pectoralis major muscle. Accordingly, since the appearance of Gray's work we have modified our technique to remove more skin, in our more recent cases skin grafting in a high proportion, and we have gradually abandoned routine sacrifice of the pectoralis major, only removing it if actually invaded, which is rare and late. The argument is sometimes used that the removal of the pectoralis major is necessary for the adequate dissection of the axilla, and that in any case its nerve supply is bound to be cut. These technical arguments are not valid. With elevation of the arm, retraction of the pectoralis major and removal of the pectoralis minor it is easy to do a complete clearance of the axillary glands and fatty tissue right up to the apex of the axilla, and at the same time to preserve the lateral pectoral nerve, the main nerve supply to the pectoralis major. Apart from this modified radical operation there are smaller series of cases which have been treated in other ways, e.g. partial mastectomy combined with axillary dissection and simple mastectomy combined with irradiation to the axilla. At the end of 1946 we decided to look up and analyse our results. In all, the Middlesex Hospital cases treated under one of us (D. H. P.) in all ways from 1930–43 amounted to 118, and we followed them up until the end of 1946, i.e. 3–17 years. The figures, particularly when subdivided into the various groups, are small, but they can be reinforced by similar experiences in private practice.

COMPARATIVE RESULTS.

*Standard radical and modified radical.*

During the period under review 45 standard radical operations were performed and 46 modified radicals, the latter term being used for the operation above described, in which skin was removed more freely but the pectoralis major preserved. The comparison between the two types of operation is best made after each group has been subdivided into cases with and cases without histological invasion of the axillary glands. Of the cases with histological invasion of the axillary glands, 26 were operated by the standard radical operation, of whom 24 were traced. Of the 24 cases with glandular invasion submitted to a modified radical operation, 22 were traced. The comparative results of the two groups are given in Table I.

TABLE I.—*Cases With Axillary Glandular Involvement.*

| | Standard radical. | Modified radical. |
|---|---|---|
| Total cases traced | 24 | 22 |
| Alive and well | 2 (6 and 11 yrs.)<br>1 (alive and well 4 yrs. then lost trace.) | 6 (3 to 7 yrs.) |
| Alive with disease | 1 (11 yrs.) | 1 (6 yrs.). |
| Died operation | 1 | 1 |
| Died other causes | 1 | 1 (4 yrs.). |
| Died disease | 18 (6 months to 7 yrs.) | 13 (1 to 8 yrs.). |

Of the cases without histological invasion of the axillary glands, 19 were operated on by the standard radical procedure, of which 18 were traced; and 20 by the modified radical, of which 18 were traced. The comparative results are given in Table II.

TABLE II.—*Cases Without Axillary Glandular Involvement.*

| | Standard radical. | Modified radical. |
|---|---|---|
| Total cases traced | 18 | 18 |
| Alive and well | 8 (4 to 14yrs.) | 13 (3 to 9 yrs.). |
| Died other causes | 2 (7 and 8 yrs.) | 0 |
| Died disease | 8 (1 to 10 yrs.) | 5 (1 to 4 yrs.). |

In addition, there were 2 cases of modified radical operations in which the condition of the axillary lymphatic glands was not noted histologically—1 died of the disease at 1 year, and 1 was alive and well at 8 years.

The results in both standard radical and modified radical are in accordance with the usual results in carcinoma of the breast in that only a minority of cases with glandular invasion live for long periods after operation, and that a considerably greater proportion so live if there has been no invasion of the axillary glands, although still a disturbingly high number die of the disease. The comparison between the figures of standard radical and the modified radical operation in this series is rendered more difficult in that the modified radical was evolved out of the standard radical, and hence has not been done for so long. The actual figures for survival in both tables are better for the modified radical, but this is vitiated by the last-mentioned fact. If, however, comparative results are reduced to the lowest common denominator of the number of cases surviving for over 3 years, a time limit useless as an indicator of absolute but valid from the point of view of comparative prognosis, a fairer comparison is obtained.

TABLE III.—*Comparison of Three Year Survival after Radical and Modified Mastectomy.*

(a) *Cases with axillary involvement.*

| | Standard radical. | Modified radical. |
|---|---|---|
| 3 yr. survivals . | 11 out of 24 traced . | 10 out of 22 traced. |

(b) *Cases without axillary involvement.*

| | Standard radical. | Modified radical. |
|---|---|---|
| 3 yr. survivals . | 14 out of 18 traced . | 15 out of 18 traced. |

It would therefore seem fair to conclude that these figures bring no evidence to support the view that the addition of the removal of the pectoralis major muscle brings any increase in the survival figures, and to raise the question whether the routine removal of this muscle should remain an essential technical point.

A further way in which the two operations can be compared is by the comparative incidence of recurrence in the operative area, i.e. skin, chest wall and axilla. Out of 42 traced standard radical operations there were 10 cases with recurrence in the operative area, chiefly skin recurrences. Of these, 7 were cases with original lymphatic glandular involvement and 3 without such involvement. Out of 40 traced modified radical operations there were 7 local recurrences. Of these, 5 were cases with original lymphatic glandular involvement and 2 without such involvement. While the incidence in both is too high, and approximates to the 22 per cent noted by Truscott in the Middlesex Hospital series, the standard radical shows no lower incidence of local recurrence than the modified.

The 3 other types of procedure which have been carried out were :

(1) In the first place there were 10 cases in which partial mastectomy was combined with the usual full axillary dissection after the removal of the pectoralis minor.

(2) Secondly, there were 9 cases in which a simple removal of the breast was combined with irradiation to the axilla.

(3) Thirdly, there were 7 cases in which irradiation only of the breast was combined with axillary dissection.

The last group comprised cases which were all advanced, the glands being invaded in all. One case died as a result of the operation; the other 6 died of the disease at intervals varying from 6 months to 4 years after treatment. Moreover, in a high proportion of cases—4 out of 6 cases which survived operation—there was either failure of the irradiation to get rid of the original primary growth in the breast, or recurrence of the growth locally after apparent disappearance. In view of the advanced character of these cases, the ultimate fatal result was likely whatever form of treatment was adopted. But the high proportion of persistence or recurrence of the growth in the breast itself led to the abandonment of this procedure. This experience confirmed that for the primary growth, at any rate, surgery is the simplest and most efficient treatment.

The 10 cases of partial mastectomy combined with dissection of the axilla were essentially early cases with small growths. Of the 2 cases with invaded glands, 1 was alive with the disease at 5 years and 1 died of other causes at 2 years. Of the 5 cases with glands not invaded, 3 were alive and well at 10 years, 9 years, and 5 years, 1 was alive with the disease at 8 years, and 1 had died of other causes 1 year after operation. There were also 3 cases in which no histological note was made of the condition of the axillary glands ; of these, 1 was alive and well at 6 years and 2 died of the disease. From the point of view of survival after operation, this group compares favourably with other groups. But the procedure has been abandoned because of the occasional further development of carcinoma in the portion of the breast remaining. This happened in 2 of the cases of this series, and it also happened in other similar cases in a private series, in spite of the fact that, as already noted, only small and apparently early cases were considered suitable for this procedure, that a wide area of breast tissue around the growth was removed, and that post-operative irradiation was given as a routine. It was concluded, therefore, that while in an occasional case partial mastectomy combined with a dissection of the axilla might be justifiable—a conclusion supporting that of Fitzwilliams (1940) and of Mitchener, Bailey and Price (1937)—the danger of further development of carcinoma in the remaining breast tissue rendered the procedure an unwise routine.

The last group was of 9 cases in which simple removal of the breast was the only surgery performed, the axilla being treated by irradiation. These cases were usually elderly patients with apparently early growths. Of the 9 cases 7 were traced. Three were alive and well at 3 years, 4 years and 8 years after operation ; 2 died of the disease, and 2 died of other causes. This procedure was abandoned, however, because it was found that the patients were more upset by the post-operative irradiation than were corresponding patients in whom the axilla was treated by surgical dissection.

Finally, there was one odd case of what appeared both clinically and histologically to be a scirrhus carcinoma of the breast in a girl aged 11 years which was treated by simple mastectomy. This patient in November, 1947 (15 years after operation), was alive, and well and about to be married.

*X-rays.*

Some of the patients after operation were treated by prophylactic irradiation and others were not. No consistent method of selection was adopted except that cases with glandular invasion were more likely to be treated. But there was no obvious difference in the results either in the gland invaded or the gland free group between those treated and those not. The absence of a regular method of selection, however, combined with the smallness of the figures renders this series unsuitable for any analysis of value on the question of the influence of prophylactic irradiation on the prognosis.

### DISCUSSION.

The high incidence of bad results in treated cases of carcinoma of the breast has led to critical consideration of the standard treatment employed, namely radical operation. While there is fairly general agreement that in most cases surgery, usually in the form of complete removal of the breast, is the simplest and most efficient way of getting rid of the primary growth, there is at the present time considerable diversity of opinion and practice on the question of what else should be done. Most surgeons still practise a standard radical operation. McWhirter (1947), following Keynes (1938), limits surgery to simple removal of the breast, treating the axilla entirely by X-rays. Handley and Thackray (1947), by demonstrating the frequency of internal mammary spread, have shown a gap in most methods of treatment of carcinoma of the breast, the best way of dealing with which is not yet settled. Our own approach has been a critical consideration of the detailed technique of the radical operation, and the results to date seem to show that routine removal of the pectoralis major in no way adds to the value of surgical treatment, thus affording some confirmation of modern views on lymphatic anatomy. The question might be rightly asked, what positive advantage is there in preserving the pectoralis major, granting that its routine removal is not necessary ? To this we would reply that the main advantages of preserving the pectoralis major are three. Firstly, there is the cosmetic consideration, mutilation being much less with the pectoralis major preserved as compared with the skeleton-like prominence of ribs and costal cartilages after its removal. And if there is no curative advantage in removal of the muscle, cosmetic considerations, although secondary, are entitled to a place. Secondly, there is less loss of blood if the muscle is not removed, and consequently less operative shock. While in most cases this does not amount to a point of much practical importance, in old or feeble patients it may. With preservation of the pectoralis major combined surgical treatment of the breast and axillary contents is, in our experience, much less upsetting than simple mastectomy combined with irradiation to the axilla, and we have as already stated for this reason largely abandoned the latter procedure. And thirdly, the pectoralis major is a much more suitable bed for skin grafting than ribs and costal cartilages. And since, in view both of theoretical lymphatic anatomical considerations and of the general (Truscott, 1947) and our own personal experiences of the undue frequency of local skin recurrences, we now attach considerable importance to a wider removal of skin, the question of the suitability of the raw area left for skin grafting becomes of practical importance.

### SUMMARY AND CONCLUSION.

1. The results are compared of different combinations of surgical and irradiation treatment of carcinoma of the breast in a personal series of 118 cases treated between 1930 and 1943, and followed up until the end of 1946.

2. Until an efficient general agent for the treatment of carcinoma of the breast is developed, a high proportion of cases are doomed to die of the disease whatever combination of local treatment by surgery and irradiation is used, because in such a high proportion of cases the disease has passed outside the field of local attack when the patient first comes for treatment.

3. A modified radical operation in which the pectoralis major is preserved shows results as good as those of the standard radical operation, and in addition has positive advantages.

4. Wider sacrifice of skin with subsequent skin grafting is desirable to reduce the incidence of local skin recurrences, which is still disconcertingly high.

5. Partial mastectomy combined with axillary dissection may be justifiable in occasional early cases with small growths, but is unjustifiable as a routine owing to the danger of local recurrence.

6. Simple mastectomy combined with irradiation to the axilla may give a satisfactory result, but the irradiation is generally more upsetting to the patient than surgical dissection of the axilla.

### REFERENCES.

FITZWILLIAMS, D. C. L.—(1940) *Brit. med. J.*, **2**, 405.
GESCHICKTER, C. F.—(1943) 'Diseases of the Breast,' Philadelphia (Lippincott).
GRAY, J. H.—(1939) *Brit. J. Surg.*, **26**, 462.
HANDLEY, R. S., AND THACKRAY, A. C..—(1947) *Brit. J. Cancer*; **1**, 15.
KEYNES, G.—(1938) *Brit. med. J.*, **2**, 302.
LANE-CLAYPON, J. E.—*Rep. Minist. Hlth. Lond.*, No. 51, 1928 ; No. 34, 1926 ; No. 28, 1924.
McWHIRTER, R.—(1947) *Lancet*, ii, 873.
MITCHINER, P. H., BAILEY, G. N., AND PRICE, A. K.—(1937) *St. Thom. Hosp. Rep.*, **2**, 192.
TRUSCOTT, B. McN.—(1947) *Brit. J. Cancer*, **1**, 129.